# Bulky Adducts in Clustered DNA Lesions: Causes of Resistance to the NER System

**DOI:** 10.32607/actanaturae.11741

**Published:** 2022

**Authors:** N. V. Naumenko, I. O. Petruseva, O. I. Lavrik

**Affiliations:** Institute of Chemical Biology and Fundamental Medicine, Siberian Branch of the Russian Academy of Sciences, Novosibirsk, 630090 Russia

**Keywords:** nucleotide excision repair, bulky DNA lesions, clustered DNA lesions

## Abstract

The nucleotide excision repair (NER) system removes a wide range of bulky DNA lesions that cause significant distortions of the regular double helix structure. These lesions, mainly bulky covalent DNA adducts, are induced by ultraviolet and ionizing radiation or the interaction between exogenous/endogenous chemically active substances and nitrogenous DNA bases. As the number of DNA lesions increases, e.g., due to intensive chemotherapy and combination therapy of various diseases or DNA repair impairment, clustered lesions containing bulky adducts may occur. Clustered lesions are two or more lesions located within one or two turns of the DNA helix. Despite the fact that repair of single DNA lesions by the NER system in eukaryotic cells has been studied quite thoroughly, the repair mechanism of these lesions in clusters remains obscure. Identification of the structural features of the DNA regions containing irreparable clustered lesions is of considerable interest, in particular due to a relationship between the efficiency of some antitumor drugs and the activity of cellular repair systems. In this review, we analyzed data on the induction of clustered lesions containing bulky adducts, the potential biological significance of these lesions, and methods for quantification of DNA lesions and considered the causes for the inhibition of NER-catalyzed excision of clustered bulky lesions.

## INTRODUCTION

**Fig. 1:**
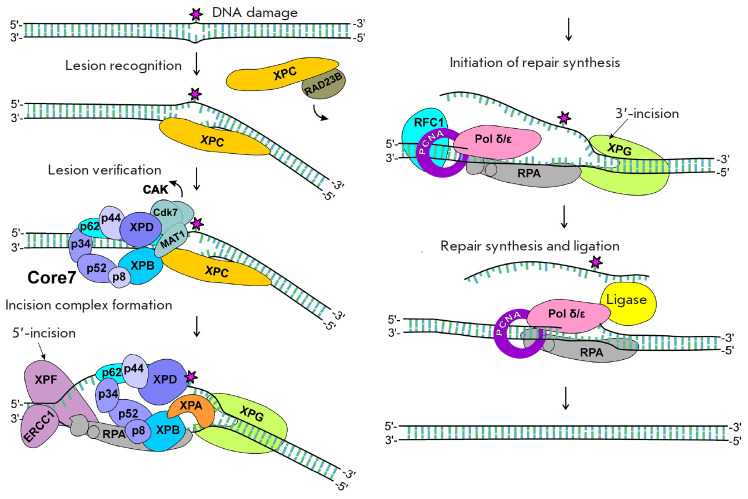
Scheme of the global genome nucleotide excision repair

The nucleotide excision repair (NER) system eliminates various DNA lesions, most of which are bulky adducts that introduce significant distortions into the regular double-stranded DNA structure. NER can be initiated via two pathways: the global genome (GG-NER) and transcription-coupled (TC-NER) ones. The transcription-coupled pathway recognizes lesions in the transcribed strands of active genes [1, 2]. TC-NER is triggered by stalling of the RNA polymerase II complex when the enzyme encounters a bulky lesion in the transcribed DNA strand. The GG-NER pathway removes lesions throughout the genome, including its non-transcribed regions and silent chromatin. In GG-NER, XPC factor complexes act as damage sensors. Starting from the second step of repair (damage verification), GG-NER and TC-NER involve the same set of protein factors and enzymes. DNA lesions are eliminated together with a 24–32-bp surrounding region. The resulting gap is filled by repair synthesis (Fig. 1) [3, 4].

Totally, NER involves more than 30 enzymes and protein factors that successively form in the DNA damage area variable on composition and structure complexes, which interact with DNA over two or three of the helix turns.

Several lesions within one or two DNA helical turns are called a clustered lesion (cluster) [5]. Clusters include various lesions: oxidized nitrogenous bases, AP sites, other non-bulky lesions, DNA strand breaks, and DNA fragments containing bulky adducts [5 , 6, 7]. In recent years, great progress has been made in understanding NER repair of single lesions [8]. In contrast, the mechanism for the removal of clustered bulky lesions is much less studied. A number of studies have shown that the formation of an additional DNA lesion near a bulky adduct often reduces the efficiency of its removal by the NER system [9, 10, 11]. In addition, simultaneous excision of lesions in the opposite DNA strands may lead to the formation of double-strand breaks that are potentially lethal for the cell [12]. On the other hand, high activity of repair systems towards induced DNA lesions in tumor cells reduces the efficiency of antitumor drugs [13, 14]. Therefore, exploration of the mechanisms of interaction between repair proteins and clustered lesions and elucidation of any relationship between the structure of therapy-induced DNA lesions and their resistance to repair is of practical importance.

In this review, we analyzed data on the formation of clustered lesions containing bulky adducts and the potential biological significance of these lesions, considered inhibition of excision of bulky DNA lesions due to NER’s unproductive binding of the XPC factor to damaged DNA, and addressed the structural features of the DNA regions containing clustered lesions resistant to NER.

## THE ORIGIN AND TYPES OF NER-REPAIRABLE DNA LESIONS

**Fig. 2:**
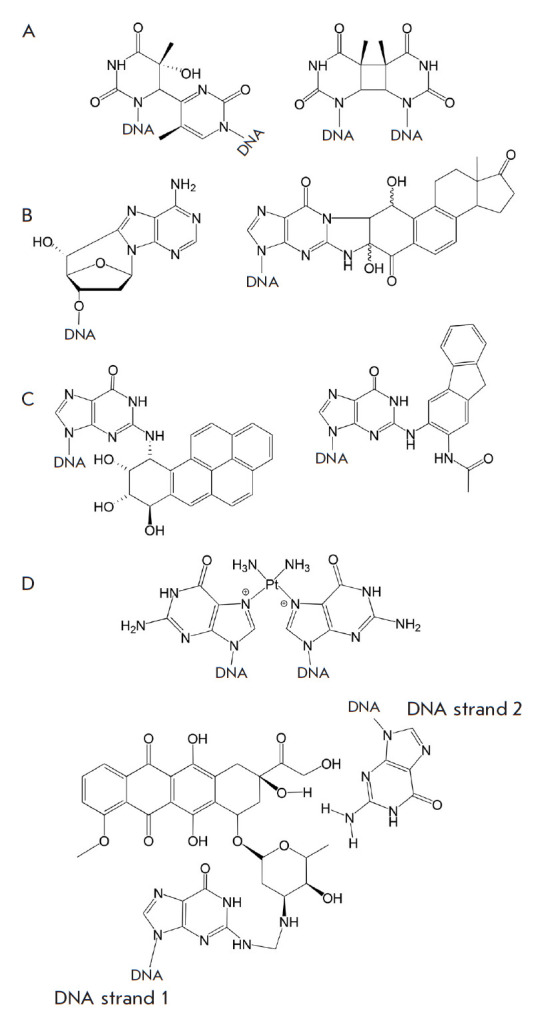
Examples of DNA lesions removed by the NER system. (*A*) UV-induced lesions: a pyrimidine-(6,4)-pyrimidine photoproduct (left) and a cyclobutene pyrimidine dimer (right). (*B*) IR-induced lesions: 8,5’-cyclo-2’-deoxyadenosine (left) and a 4-hydroxyequilenin-guanine adduct (right). (*C*) DNA modifications induced by reactive environmental molecules: a benzo[a]pyrene diol epoxide-guanine adduct (left) and a (pyridyloxobutyl)guanine adduct (right). (*D*) Chemotherapy-induced lesions: a cisplatin-DNA adduct (top) and a doxorubicin-DNA adduct (bottom)

Bulky DNA lesions, mainly covalent base adducts (Fig. 2), are induced by exposure to ultraviolet radiation (pyrimidine-(6,4)-pyrimidine photoproducts and cyclobutane pyrimidine dimers (lesion structures are shown in Fig. 2A) and strong ionizing radiation (IR) (e.g., oxidized 8,5’-cyclo-2’-deoxypurines, Fig. 2B, left; adducts of oxidized estrogen metabolites, Fig. 2B, right) [15, 16, 17]. Bulky DNA lesions are also induced by chemically active or cellular metabolism-activated substances: incomplete fuel combustion products (e.g., benzo[a]pyrene derivatives, Fig. 2C, left), tobacco smoke components (tobacco-specific nitrosamines, Fig. 2C, right [18, 19, 20]), DNA–protein crosslink-inducing agents [21], and some natural substances (e.g., aristolochic acids) [22]. Many of these lesions are difficult to repair and tend to accumulate in the body [10, 23, 24].

The cytostatic effect of many chemotherapeutic drugs is based on their ability to form bulky adducts upon interaction with DNA. These drugs include Pt-containing drugs (carboxyplatin, oxaliplatin, cisplatin; the structure of interstrand cisplatin crosslinked DNA is shown in Fig. 2D, top) [14, 25], alkylating nitrogen mustards (mechlorethamine, cyclophosphamide, and acylfulvene) [25, 26], minor groove ligands, mitomycins [27], and anthracycline drugs capable of forming covalent adducts with DNA in the presence of endogenous formaldehyde (Fig. 2D, bottom) [28].

## METHODS FOR QUANTIFICATION OF BULKY LESIONS

Quantification of DNA lesions is a challenge, because the content of damaged nucleotides in total DNA is relatively small, and their structure and properties are diverse. A wide range of methods are used to detect and quantify bulky DNA adducts. Apart from the well-known single-cell electrophoresis under alkaline conditions (alkaline DNA-comet assay) [29], there are methods based on radioactive labeling, which are characterized by limited specificity but high sensitivity to detect one adduct per 109–1010 nucleotides [30, 31, 32]. In addition, there are more selective techniques based on the use of lesion-specific antibodies (the detection threshold is one adduct per 108 nucleotides) [18, 33, 34] and new variants of the polymerase chain reaction [35]. Quantification of lesions by atomic absorption spectrometry requires a 10–50 μL sample with an expected analyte concentration of 10^–3^ to 10^–6^ M [36].

Mass spectrometric techniques provide the highest quantification accuracy and specificity for lesions. The only limitation of mass spectrometry is that acquisition of quantitative data requires the use of an isotopically labeled internal standard to allow for the formation and loss of lesions during sample processing [37, 38, 39, 40, 41].

In some cases, quantification results are discrepant, which may be due to both the imperfection of the used techniques and the structural features of the explored lesions [42]. These discrepancies are very typical of samples from patient tissues, tumor tissues, grafted tumors, cultured patient cells, and patient liquid biopsies, especially in cases of comprehensive (combination) therapy [25]. Further improvement of the methods for the quantification of DNA lesions is important both for identifying undesirable toxic effects on a living organism’s DNA and for gaining therapeutic effect data in terms of the amount of persistent DNA lesions.

## THE MECHANISMS OF INDUCTION OF CLUSTERED LESIONS CONTAINING BULKY ADDUCTS

According to rough estimates, 104–106 lesions are formed daily in the human cellular DNA [12]. Therefore, only ~0.0002–0.02% of the human genome is damaged. However, DNA lesions are nonuniformly distributed throughout the genome and are often concentrated at specific positions called mutation hotspots. Their location is indicative of both the properties of the mutation process (the predominant mutagen; efficiency of repair and replication machineries) and the structural and functional features of the cellular DNA [43].

The severity of a lesion in certain genome regions is related to many factors: the structure and amount of chemically active molecules to which the body is exposed, the mechanism of interaction between these molecules and DNA, the nucleotide sequence and local structure of DNA, and the level of chromatin compaction [43]. The small molecule–DNA interaction modes include intercalation, insertion into the minor and major DNA grooves, binding to single-stranded DNA regions, combinations of different interactions, and subsequent formation of covalent adducts with nitrogenous nucleotide bases [44].

Many substances inducing NER-repairable adducts are electrophilic compounds that interact with the nucleophilic atoms in DNA. The most reactive sites are the guanine positions N7, N2, C8, and O6; adenine positions N1, N3, and N7; thymine positions O_2_ and O4; and cytosine positions O_2_ and N4 [45]. For example, benzo[a]pyrene-7,8- diol-9,10-epoxide preferentially reacts with the guanine exocyclic (N2) amino group in the minor DNA groove. The difficult-to-repair benzo[a]pyrene adducts in this location are supposed to be the ones most often found in mammalian cellular DNA [46]. An activated aflatoxin B1 metabolite, aflatoxin B1 exo-8,9-epoxide, preferentially interacts with dG:dC-rich DNA regions and forms an adduct with (N7) guanine [47, 48]. The well-known carcinogenic aromatic amine N-2-acetylaminofluorene forms adducts at the (C8) position of guanine [49, 50]. Following metabolic activation, platinum-based chemotherapeutic agents preferentially interact with dG-rich DNA regions [51].

The risk of clustered DNA lesions significantly increases in cells under severe exposure, e.g., during intensive chemotherapy and combination therapy including exposure to radiation or additional chemotherapy drugs [5, 52, 53]. Most often, combination therapy protocols are used when essential drugs are platinum derivatives whose use is usually associated with congenital or acquired resistance. In these cases, combination therapy may include antimitotic agents terminating nucleoside analogs, topoisomerase inhibitors, and recent drugs such as paclitaxel, hemicitabine, and doxorubicin, which preferentially intercalates at the dG:dC-rich sites and forms a hydrogen bond with dG on one strand and, in the presence of formaldehyde, covalent adducts with dG on the opposite strand (Fig. 2D, bottom) [28].

Increased accumulation of oxidative lesions is characteristic of tumor [54, 55] and inflamed tissues [56]. Ionizing radiation induces DNA lesions both through direct ionization (30–40% of IR-induced lesions) and through exposure to free radicals generated during water radiolysis [57]. Exposure to γ- and X-ray radiation was found to lead to the formation of two or more AP sites, oxidized derivatives of nitrogenous bases, and DNA strand breaks within two or three turns of the DNA helix [58, 59]. Exposure to IR induces clustered lesions, such as AP sites and oxidized bases, about 4-fold more often than double-strand breaks [60, 61].

AP sites, one of the most numerous oxidative DNA lesions induced by exposure to various factors [62, 63], can exist as two forms in equilibrium: an open-ring aldehyde and a closed hemiacetal. The aldehyde form is highly reactive, which promotes the formation of additional lesions near AP sites. The reaction between the aldehyde form of an AP site and the exocyclic amino group of an adenine or guanine residue located in the opposite strand may result in dangerous DNA lesions – interstrand crosslinks (ICLs) [64]. A level of 20–40 ICLs per cell is lethal to repair-deficient mammalian cells [65]. These lesions block the separation of two DNA strands, which is required for transcription and replication. Therefore, ICLs act as absolute blockers of major cellular processes and are particularly detrimental to rapidly dividing cells. This has led to the widespread use of crosslinking agents as anticancer drugs. ICL repair pathways have not yet been definitively identified; NER proteins are believed to be involved in ICL repair in resting cells [65]. In addition, reactions of the aldehyde form of an AP site induce bulky adducts, such as intrachain crosslinks, mono-adducts, and DNA–protein crosslinks [64, 65].

The effect of radiomimetic agents, which are used in the chemotherapy of tumors, on DNA is similar to that of radiation. They promote the induction of multiple DNA lesions, such as single- and double-strand breaks and AP sites [66, 67]. One of these agents is bleomycin, a glycopeptide with pronounced cytotoxic and mutagenic properties, which is produced by Streptomyces verticillus bacteria. One part of the bleomycin molecule binds to the minor DNA groove and modifies its nitrogenous bases, while the other part is able to react with metal ions (e.g., Fe (II)) and oxygen and form reactive oxygen species that induce additional oxidative lesions in the adjacent DNA regions [66, 68].

Induction of clustered lesions is also affected by the accessibility of specific DNA regions to a damaging agent. Chromatin proteins protect DNA from the damaging effects of IR, free radicals, and genotoxic chemical compounds [69, 70, 71].

On the contrary, bulky lesions induce a significant local weakening of the Watson-Crick interactions and, thus, facilitate the accessibility of DNA to oxidative and other damaging agents and increase the likelihood of spontaneous glycosidic bond hydrolysis and AP site formation. Therefore, the presence of spontaneous or induced bulky adducts increases the risk of clustered lesions in the surrounding DNA region [62, 72]. For example, exposure of DNA containing platinum adducts to even low radiation doses was shown to increase the risk of clustered lesions 1.5 to 2.5-fold [73, 74]. Given that clustered lesions are often difficult to repair, this exposure during combination therapy may promote the accumulation of platinum adducts in the DNA of cancer cells, despite the fact that, in some cases, cancer cells are characterized by an increased activity of DNA repair systems [75, 76].

## RECOGNITION OF DNA LESIONS BY GLOBAL GENOME NER

During the global genome NER process, the primary recognition of a DNA region containing a bulky lesion occurs without direct contact between the XPC sensor protein and the lesion [3, 77, 78]. As already noted, bulky lesions induce changes in the regular dsDNA structure, which are often accompanied by a destabilization of the molecule and the formation of mobile single-stranded regions with increased affinity for XPC. During the search for lesions, XPC moves along the DNA molecule in a repeated association-dissociation manner, forming many short-lived complexes with DNA, which allows XPC to bypass obstacles: proteins associated with DNA [79].

**Fig. 3:**
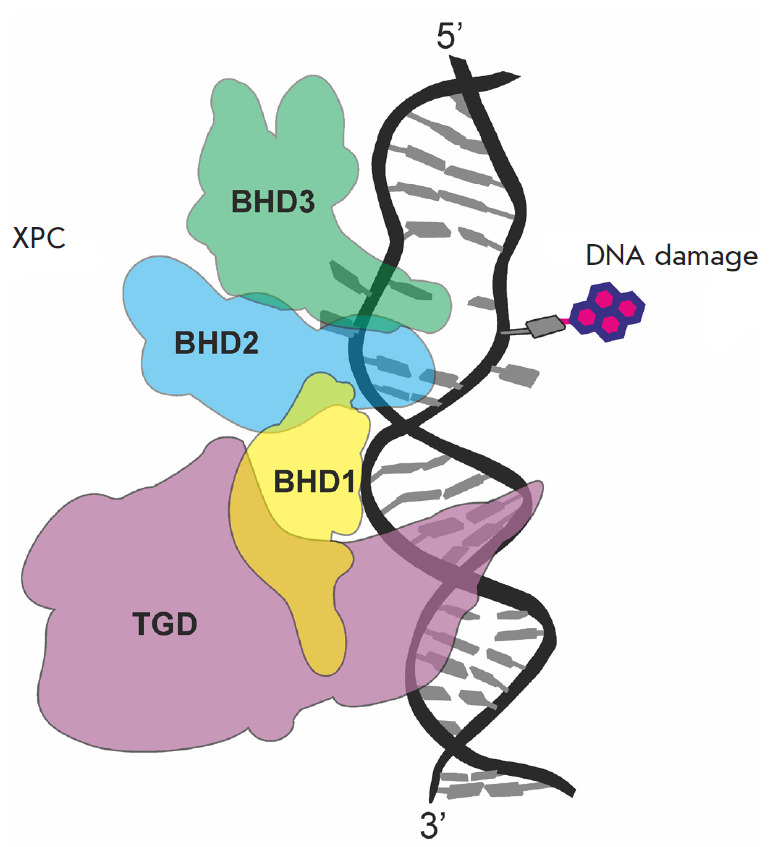
DNA damage recognition by the XPC protein. DNA damage (pink), the transglutaminase (TGD) domain of XPC (purple), the BHD1 domain (yellow), the BHD2 domain (blue), and the BHD3 domain (green)

A more detailed understanding of the first NER step has been gained from biochemical experiments, such as photoaffinity modification and steady-state fluorescence titration with a recombinant human XPC protein and its yeast orthologue Rad4, as well as X-ray diffraction analysis of the Rad4 protein associated with DNA containing a cyclobutane pyrimidine dimer [77, 80]. XPC comprises three β-hairpin domains: BHD1, BHD2, and BHD3 (Fig. 3) [77]. At the first step of lesion recognition, the BHD1 and BHD2 domains of the XPC factor recognize DNA regions with weakened hydrogen bonds. Regions with a weakened regular DNA duplex structure are recognized via sequential interactions between an aromatic sensor (the amino acid residues Trp690 and Phe733 located in the BHD2 domain) and aromatic heterocyclic nitrogenous bases [81, 82]. The XPC subunit comprising the aromatic sensor is similar to the oligonucleotide/oligosaccharide-binding motif typical of proteins that preferentially interact with single-stranded DNA; e.g., RPA [81, 82, 83]. The BHD1 and transglutaminase domains of XPC bind to an 11-bp segment of undamaged DNA at the 3’- end of the lesion, harboring the protein from DNA [82].

Then, a more specific XPC–DNA complex is formed in the immediate vicinity of the lesion. In this complex, two β-hairpin domains, BHD2 and BHD3 (Fig. 3), interact with a 4-nucleotide segment of the undamaged strand, which is located opposite the lesion (Fig. 3) [77, 84]. Structural studies of a complex between the yeast orthologous protein Rad4 and damaged DNA [77] revealed that binding of BHD2/3 results in the extrusion of both the damaged nucleotide and two undamaged nitrogenous bases in the complementary strand from the DNA duplex that occurs in a flipped-out open conformation. A long β-hairpin protruding from BHD3 is inserted into DNA, thereby stabilizing the structure formed during nucleotide flipping-out. In this case, the DNA backbone is kinked by about 40°. An XPC–DNA complex of a specific structure is formed, which involves a rather extended DNA region near the lesion (Fig. 3).

The selectivity of a search for lesions is controlled by a ratio of the time of DNA–XPC complex formation and its lifetime. Usually, NER-productive complexes are characterized by a shorter formation time and an optimal lifetime [85, 86]. Calculations performed using a model of stochastic reversible nucleoprotein NER complex formation revealed that the initial recognition of a lesion-containing DNA region is the slowest NER step that limits the rate of lesion removal [87]. The efficiency of the first NER step, recognition of damaged bases in a huge intact DNA, controls the rate of the entire repair process [85, 88, 89].

In the cell, XPC occurs as XPC–RAD23B and XPC–RAD23B–Cen2 complexes. The RAD23B subunit stabilizes the XPC protein and promotes its interaction with DNA. Following XPC binding to a damaged DNA region, the RAD23B subunit dissociates from the complex. The function of the Cen2 subunit in these complexes is not fully understood; in vitro, it is not required for NER [90]. However, it is known that Cen2, although not in contact with DNA, stimulates NER as a whole and is required for effective recruitment of the TFIIH factor to the repair process [91, 92].

Following the initial step of lesion recognition and XPC–DNA complex formation, a bulky DNA lesion is verified by the TFIIH factor. The TFIIH complex comprises a seven-subunit core (Core7), which is composed of the ATP-dependent helicases XPB and XPD and non-enzymatic subunits p62, p52, p44, p34, and p8, and the so-called CDK-activating kinase (CAK) complex that involves the MAT1, cyclin H, and Cdk7 subunits [93, 94]. In the presence of the CAK complex, XPB, and XPD subunits are connected via a long α-helix of the MAT1 protein, with TFIIH being in a rigid ring-like conformation that limits their enzymatic activity. After recruitment of TFIIH to NER, the CAK heterotrimer is released from the complex and Core7 forms a more flexible horseshoe-shaped structure, with XPB and XPD being located at each end of the horseshoe (Fig. 1) [8, 95].

Core7 binds to the repair complex through the interaction between its XPB and p62 subunits and the XPC factor associated with a damaged DNA region [96, 97]. The interaction between the XPB subunit and the XPC C-terminus located at the 5’- end from the lesion stimulates the ATPase activity of XPB and leads to the conformational rearrangement of Core7 and its binding to a DNA substrate [98, 99]. This conformational rearrangement enables XPD to bind to the damaged DNA strand on the 5’- side from the lesion.

XPD acts as a molecular sensor that verifies a bulky lesion in a DNA strand. Due to the 5’-3’-helicase activity stimulated by the p44 subunit, the protein moves to the lesion and forms an asymmetric bubble. During XPD activity, the damaged strand passes through a pore formed by the FeS, Arch, and HD1 domains of XPD and each base of the strand comes into contact with a sensor pocket on the protein surface. When XPD encounters damage, its helicase activity is inhibited and XPD is immobilized on DNA, thus marking the damage for its subsequent removal by the proteins of the incision complex (Fig. 1) [100, 101].

## THE INFLUENCE OF THE DNA STRUCTURE ON THE REPAIR OF CLUSTERED BULKY LESIONS

**Fig. 4:**
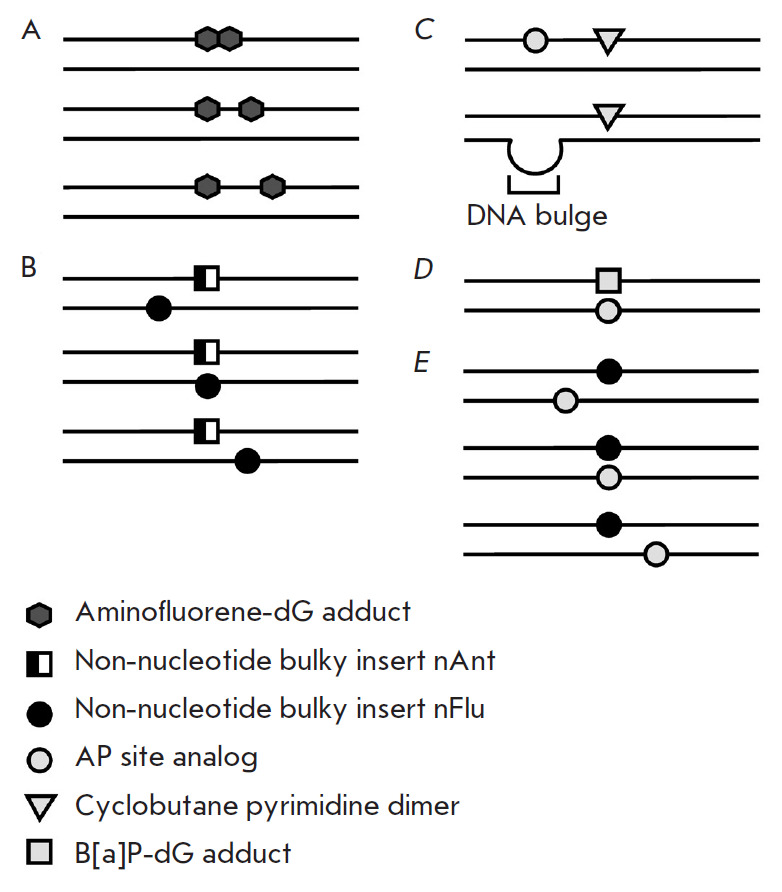
Schematic of model DNAs containing clustered lesions. (*A*) Circular plasmid DNA containing fluorinated aminofluorene mono- or di-adducts separated by one or two nucleotides. (*B*) DNA containing synthetic bulky lesions in both strands: non-nucleotide inserts containing a bulky anthracenylcarbamoyl (nAnt) or fluorescein carbamoyl (nFlu) residue; the length of a model DNA duplex is 137 bp; the interlesion distance is ≤ 20 bp. (*C*) DNA duplexes (~200 bp) containing CPD and an AP site analog in the same strand or CPD and a bulge in the com plementary strand. (*D*) A 135-bp DNA duplex containing a benzo[a]pyrene diol epoxide-guanine adduct and an opposite AP site analog. (*E*) DNA containing an nFlu bulky lesion and an AP site analog in the opposite strand; the length of a model DNA duplex is 137 bp; the inter lesion distance is ≤ 6 bp

Significant progress in understanding the recognition and removal of clustered lesions by the NER system has been achieved thanks to in vitro studies using synthetic oligodeoxyribonucleotides with lesions at specified positions of DNA strands [10, 11, 102]. Figure 4 presents a schematic of DNA containing clustered lesions of various structures: in particular natural and synthetic bulky lesions used in these studies.

There is a direct correlation between the efficiency of the repair of some bulky DNA lesions by NER and the affinity of the XPC–RAD23B factor for these DNAs: e.g., single aminofluorene adducts located in DNA of the same sequence [88]. However, increased affinity of XPC–RAD23B for bulky lesion-containing DNAs is not always associated with a high efficiency of their excision in the case of both single, bulky lesions and clustered lesions [10, 11, 63, 86, 103]. For example, a benzo[a]pyrene adduct, R-cis-B[a]P-dG, is removed by NER proteins 5-fold more efficiently than the S-trans-B[a]P-dG isomer, despite the fact that the affinity of XPC–RAD23B for the corresponding DNA duplexes is the same [103]. Also, with minimal differences in the affinity of XPC-RAD23B for DNA with single, synthetic lesion analogs nAnt (a non-nucleotide insert with a bulky anthracenylcarbamoyl substituent) and Fap-dC (cytosine with a fluoro-chloro-azidopyridyl group introduced at the exocyclic nitrogen), the former lesion is repaired by NER proteins, while the latter is unrepairable [104].

DNAs containing clustered lesions, in particular bulky adducts, are usually characterized by increased affinity of the XPC factor for them. However, repair of such lesions by NER is partially or completely inhibited in many cases [10, 11, 63]. In [86], the real-time monitoring surface plasmon resonance technique was used to investigate interactions between XPC–RAD23B and DNAs containing single and cluster adducts formed by active metabolites of a fluorinated acetylaminofluorene derivative and C8 guanine. These adducts, forming a clustered lesion, were located in the same DNA strand and were separated by two or fewer nucleotides (Fig. 4A). The XPC factor was shown to form significantly more stable complexes with DNA containing clustered lesions compared with DNA containing single acetylaminofluorene adducts. In this case, NER excision activity towards DNAs with clustered lesions was lower than that towards DNAs with single lesions (in some cases, it was lower than the detection ceiling). Inhibition of specific excision in this case is supposed to be the result of disturbances in the assembly of the protein complexes responsible for the verification of DNA damage, which is due to extremely strong binding of XPC to the damaged site [86]. At KD values of 10^–11^–10^–12^ M, the XPC factor can compete for binding even with a single-stranded DNA sensor, the RPA protein, which, to gether with XPA, is part of the NER pre-incision and incision complexes [3, 105 , 106, 107].

NER activity is also hindered by synthetic lesion analogs located in both DNA strands, whose bulky fragments are connected to the DNA backbone by extended flexible linkers (Fig. 4B) [102]. These linkers allow bulky aromatic groups of adducts to come into contact with the DNA regions adjacent to the lesion, which may induce additional destabilized DNA regions that stimulate XPC binding. A site with weakened Watson–Crick pairing near the DNA damage is supposed to be able both to inhibit and to enhance the efficiency of NER, depending on its location. The presence of this destabilization site on the 3’-side from the lesion may induce a DNA–XPC complex unproductive for subsequent NER steps: in this case, the encounter of TFIIH with the lesion is excluded [104, 108, 109, 110]. On the contrary, a DNA destabilization site on the 5’-side from the lesion may stimulate the NER process. For example, introduction of an AP site analog shifted relative to the CPD position towards the 5’-end of a damaged DNA strand was shown to stimulate excision of the CPD-containing fragment by NER [111]. A bulge in the DNA duplex on the 5’-side from CPD also increases the efficiency of its excision manifold (model DNAs are schematically shown in Fig. 4C). The observed effects are also associated with the features of the mechanism of lesion recognition by the TFIIH factor; namely, with the 5’-3’-direction of its movement along DNA from the primary binding site and strand unwinding direction.

Of particular interest is the investigation of the repair mechanism of clustered lesions composed of bulky DNA adducts and oxidative lesions to DNA nitrogenous bases [10, 11]. As mentioned above, a DNA region destabilized by a bulky lesion is more susceptible to reactive oxygen species, thus increasing the risk of clustered lesions. These clustered lesions can attract nucleotide excision repair and base excision repair (BER) proteins.

The repair of a clustered lesion composed of a bulky B[a]P adduct and an AP site analog, which are located in the complementary strands of the DNA duplex, was analyzed in [10] (model DNA is shown in Fig. 4D). Evaluation of the NER excision activity towards B[a]P-dG and the ability of AP endonuclease 1 to hydrolyze the AP site showed that NER was inhibited in these clusters, while the AP sites were repaired by BER. Therefore, the NER system is sensitive to oxidative AP site-like lesions in the immediate vicinity of B[a]P-dG [8, 10]. A further detailed study of the interaction between this model structure and repair proteins revealed that XPC stimulated the endonuclease activity and inhibited the 3’-5’-exonuclease activity of AP-endonuclease 1, thereby increasing the efficiency of BER [63].

Liu et al. [10] used NMR spectroscopy, measurements of the DNA duplex thermal stability, and computer simulation to demonstrate that DNA containing an AP site opposite a B[a]P-dG adduct is characterized by strong stacking interactions between B[a]P aromatic rings and neighboring nitrogenous bases of the complementary strand, which may inhibit XPC–DNA complex formation. In this case, the flipping of neighboring nucleotides, insertion of a β-hairpin of the BHD3 domain, and extrusion of the lesion from the DNA helix are impeded. Moreover, the XPC factor was characterized by increased affinity for the tested DNAs a containing clustered lesion [63].

A benzo[a]pyrene adduct also became unrepairable by NER upon deletion of its complementary dC nucleotide. NMR spectroscopy and computer simulation studies [9, 112] demonstrated that deletion of dC significantly enhances stacking interactions between the B[a]P aromatic ring and the surrounding nitrogenous bases, which prevents the formation of a productive open XPC–DNA complex [112].

Naumenko et al. [11] explored the effect of an AP site analog located on different sites of the complementary DNA strand on the removal of a non-nucleotide insert comprising a bulky fluorescein carbamoyl fragment (nFlu) by NER (Fig. 4E). The XPC factor and DNA formed unproductive complexes in which the nFlu bulky lesion and the AP site analog were separated by less than 6 bps. There was an inverse correlation between the relative efficiency of excision of nFlu-containing fragments from these model DNAs and the affinity of XPC for the model DNAs. The location of the AP site and nFlu in opposite positions of the DNA duplex, as well as similar localization of other lesions (B[a]P-dG/AP site, nAnt/nFlu), completely inhibited the excision of the bulky damage by NER proteins (Fig. 4 B, D) [10, 102].

Structural DNA changes associated with inhibited nFlu excision in the presence of an AP site analog in the complementary strand (Fig. 4E) were revealed using molecular dynamics. Simulation of molecular dynamics trajectories showed that DNA with nFlu and an AP site analog, which were located opposite each other in the complementary strands, was in a “compressed” conformation of the duplex at the lesion site: the bases adjacent to the lesion were characterized by effective stacking interactions with each other most of the time, and both lesions were flipped out of the strands. The fluorescein moiety (Flu) occurred in the minor groove, oriented towards the 5’-end of the damaged strand, which had the potential to sterically hinder binding of XPC to a destabilized DNA region located on the 5’-side from the lesion. In this case, an unproductive XPC binding site on the 3’-side from nFlu became more accessible [11], which has the potential to lead to the formation of an XPC–DNA complex unproductive for NER. For a short time, Flu may be oriented towards the 3’-end of the damaged strand and interact with the AP site analog on the opposite side of the DNA helix.

Therefore, an additional non-bulky lesion of a nitrogenous base (e.g., an AP site) or a deletion in the complementary strand, opposite a bulky DNA adduct, may induce local stabilization of the damaged site [9 , 10, 11, 112], which prevents binding of the XPC factor, thus excluding the subsequent NER steps.

The verification step may also affect the efficiency of NER lesion removal. Affinity of XPD for model DNAs containing single bulky lesions with similar XPC affinity (KD = 1.5–3 nM) was recently shown to depend on the structure of bulky lesions and vary significantly, being correlated with the efficiency of lesion removal in vitro [104, 113]. The number of studies on the verification of clustered DNA lesions is rather small. For example, introduction of an AP site into a DNA substrate (either into the strand scanned by XPD helicase or into the complementary strand, “invisible” for XPD) was shown not to significantly affect the helicase and ATPase activity of recombinant Core7 [106]. Thus, the verification step is unlikely to promote significant differences in the efficiency of NER in DNAs containing clustered lesions of this composition.

It should be noted that obstacles to a successful repair of a bulky adduct from a clustered lesion may also include steric hindrances during excision of a damaged DNA fragment by the XPF and XPG endonucleases and the lack of an undamaged DNA template of the complementary strand. However, this topic has not been well addressed and requires further research.

## CONCLUSION

Due to differences in the chemical properties of nitrogenous DNA bases and the type and strength of genotoxic factors, lesions are unevenly distributed over cellular DNA, concentrating in certain regions of the genome. Clustered lesions are often difficult to repair, which leads to their accumulation in DNA, especially if the repair status of the cell is reduced. On the other hand, hindered DNA repair of induced lesions should promote their cytotoxic effect on cancer cells. Model DNA studies have shown that removal of bulky lesions during global genome NER may be inhibited at the initial recognition step due to the structural features of a cluster-containing DNA region. In the clustered DNA lesions formed by a bulky adduct and an opposite AP site, the AP site was shown to be processed by BER enzymes rather efficiently, while NER excision of a bulky lesion from these structures was difficult. Sequential removal of lesions from clusters is supposed to be of adaptive value, because it excludes simultaneous initiation of NER and BER. Understanding the mechanisms of removal of clustered DNA lesions containing bulky adducts should help develop rational and efficient approaches to the maintenance of therapy-induced DNA lesions in cancer cells with increased activity of DNA repair systems.

## Abbreviations

AP site: apurinic/apyrimidinic site
B[a]P-dG: benzo[a]pyrene-guanine adduct
BER: base excision repair
BHD: β-hairpin domain of the XPC protein
CPD: cyclobutane pyrimidine dimer
ICL: interstrand DNA crosslink
IR: ionizing radiation
nAnt: non-nucleotide fragment of a DNA strand containing a bulky anthracenylcarbamoyl residue
nFlu: non-nucleotide fragment of a DNA strand containing a bulky fluorescein residue
NER: nucleotide excision repair
